# Genetic variation in interleukin-7 is associated with a reduced erythropoietic response in Kenyan children infected with *Plasmodium falciparum*

**DOI:** 10.1186/s12881-019-0866-z

**Published:** 2019-08-16

**Authors:** Lily E. Kisia, Prakasha Kempaiah, Samuel B. Anyona, Elly O. Munde, Angela O. Achieng, John M. Ong’echa, Christophe G. Lambert, Kiprotich Chelimo, Collins Ouma, Douglas J. Perkins, Evans Raballah

**Affiliations:** 1 Department of Biomedical Sciences and Technology, School of Public Health and Community Development, Maseno University, Maseno, Kenya; 2 University of New Mexico/KEMRI Laboratories of Parasitic and Viral Diseases, Kisumu, Kenya; 3 Department of Internal Medicine, Center for Global Health, University of New Mexico Health Sciences Center, Albuquerque, NM USA; 4 Department of Medical Laboratory Sciences, School of Public Health, Biomedical Sciences and Technology, Masinde Muliro University of Science and Technology, P.O. Box 190-50100, Kakamega, Kenya

**Keywords:** *IL7* variation, *Plasmodium falciparum*, Severe malarial anemia, Inefficient erythropoiesis

## Abstract

**Background:**

Severe malarial anemia (SMA) is a leading cause of malaria-related morbidity and mortality in children. The genetic factors that influence development of SMA and inefficient erythropoiesis, a central pathogenic feature of SMA, are only partially understood.

**Methods:**

We performed a pilot Genome-wide Association Study (GWAS) on children with *Plasmodium falciparum*. The GWAS was performed using the Illumina® Infinium® HD Super Assay in conjunction with Illumina’s® Human Omni2.5-8v1 BeadChip (with > 2.45 M markers). Data were analyzed using single SNP logistic regression analysis with an additive model of inheritance controlling for covariates. Results from our pilot global genomics study identified that variation in interleukin (IL)-7 was associated with enhanced risk of SMA. To validate this finding, we investigated the relationship between genotypes and/or haplotypes of two single nucleotide polymorphisms (SNPs) in *IL7* [72194 T/C and − 2440 A/G] and susceptibility to both SMA and inefficient erythropoiesis [i.e., reticulocyte production index (RPI) < 2.0 in anemic children (Hb < 11.0 g/dL). Children presenting with *P. falciparum *malaria (< 3 years, *n* = 883) were stratified into two groups: Uncomplicated malaria (UM, *n* = 718) and SMA (*n* = 165).

**Results:**

Regression modeling, controlling for anemia-related confounders, revealed that carriage of the TC genotype at position 72194 T/C was associated with enhanced susceptibility to inefficient erythropoiesis (OR = 1.90; 95% CI 1.09–3.30; *P* = 0.02) as was homozygous CC (OR 5.14; 95% CI = 1.20–21.99; *P* = 0.03). Consistent with this finding, individuals with the CA (72194C/−2440A) haplotype had an increased risk of inefficient erythropoiesis (OR = 1.90; 95% CI = 1.10–3.30; *P* = 0.02), whereas TA haplotype carriers had marginal protection against inefficient erythropoiesis (OR = 0.24; 95% CI = 0.06–1.21; *P* = 0.05). These observations were supported by Cochran-Armitage trend test for inefficient erythropoiesis (CA > TA > CG; *P* < 0.01). Although none of the genotype and/or haplotypic variants were significantly associated with SMA, the direction of the risk profiles were consistent with the erythropoiesis results.

**Conclusion:**

Taken together, variation in *IL7* is associated with erythropoietic responses in children with falciparum malaria, a central physiological feature contributing to development of SMA.

**Electronic supplementary material:**

The online version of this article (10.1186/s12881-019-0866-z) contains supplementary material, which is available to authorized users.

## Background

The majority of malaria-related deaths occur in Sub-Saharan Africa as a consequence of infection with *Plasmodium falciparum* [1]. Severe malarial anemia [SMA, hemoglobin, (Hb<5.0 g/dL), with any density parasitemia], is the most prevalent life-threatening complication of severe malaria occurring in infants and young children in holoendemic *P. falciparum* transmission areas [1]. The etiology of SMA involves direct and indirect destruction of parasitized and non-parasitized red blood cells (RBCs), and reduced erythropoiesis [2]. We previously demonstrated that genetic variation in immune response genes (i.e., cytokines, chemokines, and effector molecules) influence susceptibility to SMA by altering the production of inflammatory mediators [3–8]. To identify novel targets that influence susceptibility to SMA, we utilized a global genomic approach [i.e., Genome-wide Association Study (GWAS)] which identified interleukin (IL)-7 as a potential candidate locus for further validation (unpublished data).

IL-7 is a hematopoietic growth factor which modulates both T- and B-cell development and T-cell homeostasis [9]. The effects of IL-7 are mediated through binding to both the IL-7 receptor alpha (IL-7Rα) and common cytokine gamma chain (γc) which is shared with members of the IL-2 cytokine family (i.e., IL-2, IL-4, IL-9, IL-15, and IL-21) [10]. Production of IL-7 is primarily from non-hematopoietic cells such as stromal cells in the bone marrow and thymus, dendritic cells, hepatocytes, keratinocytes, and epithelial cells [11, 12]. IL-7 induces differentiation of multipotent (pluripotent) hematopoietic stem cells into lymphoid progenitor cells, promotes proliferation, differentiation, and survival of naïve and mature T cells, and stimulates the production of myelopoietic factors (e.g., IL-3 and GM-CSF) [11, 12]. Additionally, IL-7 up-regulates T-cell-dependent activation of monocytes/macrophages which initiates the production of tumor necrosis factor (TNF)-α, IL-1, IL-6, IL-8, and macrophage inflammatory protein-1β (MIP-1β) [9, 13]. Importantly, IL-7 also mediates cell-mediated immune responses to human pathogens, and induces tissue destruction in inflammatory diseases [9, 13–17].

Human *IL7* is located on chromosome 8q12–13 and contains 6 exons distributed over more than 33-kbp [18]. In non-African populations, variation in the *IL7* locus has been shown to influence susceptibility to inflammatory diseases, including multiple sclerosis and osteoarthritis [19–21]. In addition, a targeted genetic-association study in pregnant women from Mozambique revealed that *IL7* was strongly associated with susceptibility to placental malaria [22]. A role for IL-7 in malaria is also suggested by studies in murine models in which malarial anemia was associated with suppression of erythropoietic-related cytokines [i.e., granulocyte colony stimulating factor (G-CSF), GM-CSF, IL-7, and IL-17], and elevation of IL-10 and TNF-α [23]. These results are similar to our previous findings showing that levels of circulating IL-7 are suppressed in children with SMA [24].

We postulate that reduced peripheral levels of IL-7 are contributing to inefficient erythropoietic responses. This hypothesis is consistent with results in mice infected with *P. yoelii* showing that levels of erythropoietic-related cytokines, including IL-7, G-CSF, GM-CSF and IL-17 were reduced in the context of malarial anemia, relative to anemia by other causes [23]. Based on the potentially important role of IL-7 in malaria, results presented here examined the relationship between two *IL7* variants identified in the pilot genomic wide experiment (72194 T/C, rs2583759 and − 2440 A/G, rs7007634) and susceptibility to SMA in Kenyan children. In addition, we explored the association between the *IL7* variants and inefficient erythropoiesis [defined by a reticulocyte production index (RPI), < 2.0].

## Methods

### Study participants

Children with falciparum malaria (aged < 3 years, *n* = 883) presenting at Siaya County Referral Hospital (SCRH) for their first documented visit for acute malaria were recruited. Siaya County is a rural area in western Kenya with holoendemic *P. falciparum* transmission largely inhabited by Luo ethnic tribe (> 96%), thus, providing a homogeneous population for genetic-based studies. The study site for the current investigation is described in detail in our previous report [25]. Written informed consent in the language of choice (Dholuo, Swahili or English) was sought from the parent/guardian of each child participating in the study. A questionnaire was used to collect demographic and clinical information (Additional file 3). Children were excluded from the study if they had a positive blood smear with non-*P. falciparum* species; any previous hospitalization; and/or a diagnosis of cerebral malaria. Hemoglobin (Hb) concentrations were used to stratify parasitemic children into SMA (Hb < 5.0 g/dL; *n* = 165) and uncomplicated malaria [UM (Hb ≥ 5.0 g/dL; *n* = 718)] [1].

Since nearly all children in holoendemic regions, such as Siaya, develop repeated episodes of malaria prior to reaching naturally-acquired immunity, and it is unclear what disease outcome will occur in healthy children once they acquire malaria, UM was selected as the control group in the study. All children were tested for absence/presence of HIV-1 and bacteremia since we have previously shown that these co-infections impact on malarial anemia severity [8, 26]. Pre- and post-test HIV counselling was provided to the parents/guardians of the study participants. The study was approved by the Scientific Ethics and Research Committee (SERU) of the Kenya Medical Research Institute (KEMRI) and University of New Mexico Institutional Review Board. All samples were collected prior to the administration of any treatment regimen. Patients were treated according to the Ministry of Health (MOH)-Kenya guidelines.

### Laboratory procedures

Venous blood samples (<3.0 mL) were collected in EDTA-containing vacutainer tubes. Trophozoites were counted against 300 leukocytes in peripheral blood smears stained with 10% Giemsa for 15 minutes. Parasite density was estimated using the formula: parasite density/μL=white blood cell (WBC) count/μL*trophozoites/300. Complete hematological parameters were determined using a Beckman Coulter® AcT diff^2^™ (Beckman–Coulter Corporation). Reticulocytes were stained using new methylene blue on thin blood films and then counted. The reticulocyte production index (RPI) was calculated as follows: RPI=reticulocyte index (RI)/maturation factor (MF), where RI=(reticulocyte count [%] x hematocrit [Hct]/0.36) and MF=b+(*m*)(*x*), where *b*=1, *m*=0.05, and *x*=(average normal population Hct - patient’s Hct) [27]. Typing for the common African 3.7-kb α-globin deletion (α-thalassemia) was carried out by PCR as previously described [8]. *Hemoglobin* phenotypes were determined by cellulose acetate electrophoresis, while glucose-6-phosphate dehydrogenase (G6PD) deficiency was determined through the fluorescent spot test (Sigma-Aldrich) as previously described [4, 8]. HIV infection was assessed by HIV-1 proviral DNA PCR testing according to our published methods [26]. Bacteremia was determined using Wampole Isostat Pediatric 1.5 system (Wampole Laboratories). API biochemical galleries (bioMerieux, Inc.) and/or serology were used for identification of bacterial isolates [28].

### High-throughput genotyping

A pilot GWAS case-control study was performed in children infected with falciparum malaria to identify novel genes/gene pathways involved in the pathogenesis of SMA. Children were stratified into polarized phenotypes: SMA (cases, avg. Hb = 4.1 g/dL, *n* = 70) and UM (controls, avg. Hb = 10.8, *n* = 74) to enrich the signals in the tails (extremes) of the disease distribution. Selected individuals were from a cohort of children infected with *P. falciparum* (*n* = 1218), and matched according to age, sex, and peripheral parasite density, excluding children with HIV-1, bacteremia, sickle-cell disease (SSD), and G6PD deficiency. The GWAS was performed using the Illumina® Infinium® HD Super Assay in conjunction with Illumina’s® Human Omni2.5-8v1 BeadChip (with > 2.45 M markers). Data were analyzed using single SNP logistic regression analysis with an additive model of inheritance, while controlling for covariates. Analysis was performed using SNP & Variation Suite v8.6 (Golden Helix, Inc., Bozeman, MT, http://www.goldenhelix.com).

### Transcriptional factor binding sequence analysis

Polymorphic variation in the promoter regions can functionally affect the transcriptional activity of genes [29]. To determine either gain or loss of binding for (potential) transcription factors (TFs) due to SNP variation, we performed *in-silico* transcription factor binding site (TFBSs) analyses on the region encompassing the promoter variant using TRANSFAC [30, 31]. Specifically, analysis of *IL7*–2440 A/G (rs7007634)] was carried out to identify potential changes in TFBS.

### Interleukin-7 genotyping

Genomic DNA was extracted using the MasterAmp™ Buccal swab DNA extraction kit (Epicentre Biotechnologies, Madison, WI), and amplified using the Genomiphi DNA amplification kit (GE Health, Life Sciences, Amersham). *IL7* (72194 T/C, rs2583759 and − 2440 A/G, rs7007634) was genotyped by a TaqMan 5′ allelic discrimination Assay-By-Design method according to the manufacturer’s instructions (Assay ID: C_9168017_10 for 72194 T/C and Assay ID: C_30930344_10 for − 2440 A/G; Applied Biosystems, Inc.). PCR was performed in a total volume of 10 μL with the following amplification protocol: initial denaturation (60 °C for 30s and 95 °C for 10 min.) followed by 40 cycles of (95 °C for 15 s and 60 °C for 1 min.) and a final extension (− 60 °C for 30s). Genotypes were then determined using allele-specific fluorescence on the StepOnePlus™ Real-Time PCR Systems. StepOne™ Software Version 2.3 was used for allelic discrimination (Applied Biosystems, Inc.). Genotypes were then used to generate haplotypes in HPlus software (Fred Hutchinson Cancer Research Center, Seattle, WA, USA).

### Statistical analyses

Statistical analyses were performed using SPSS (version 19.0). Differences between proportions and comparisons of genotype and haplotypic frequencies of *IL7* polymorphisms between the UM and SMA groups were performed using the chi-square (χ^2^) tests. Mann-Whitney U test was used for comparisons of demographic, clinical, and laboratory characteristics between the clinical groups. The relationship between *IL7* genotypes/haplotypes, SMA, and inappropriate erythropoiesis were determined using bivariate logistic regression in a model that controlled for potential confounding variables [age, gender, HIV-1 and bacteremia status, Hb phenotypes, G6PD deficiency, and α-thalassemia variants]. Statistical significance was set at *P*≤0.05. As secondary analyses, multiple linear regression modeling was performed with RPI as the outcome variable. For this model, identical covariates as those used in the bivariate models were entered into block 1, while and genotypes/haplotypes were entered into block 2. In addition, the Cochran-Armitage trend test was used to establish trends in disease outcomes among the genotypes/haplotypes.

## Results

### Characteristics of the study participants

The demographic, clinical, and laboratory characteristics of the study participants are presented in Table 1. Gender was comparable between the two clinical categories (*P*=0.72). Children with UM and SMA differed by age, with the UM group being older than children with SMA (*P*=0.01). Axillary temperature differed between the two clinical groups (*P*<0.01). As expected based on the *a priori* grouping, hemoglobin (Hb) concentrations and red blood cell counts (RBC) were lower in SMA patients in relation to the UM group (*P*<0.01 and P<0.01, respectively). Red cell distribution width (RDW), mean corpuscular hemoglobin concentration (MCHC), and reticulocyte production index (RPI) were comparable between the clinical groups (*P*=0.48, *P*=0.79 and *P*=0.42, respectively). Inefficient erythropoiesis (RPI<2.0) was marginally lower in children with SMA (*P*=0.08). The RPI is used as marker in the diagnosis of anemia due to altered erythropoietic responses. This standard measure corrects for both the degree of anemia, and the early release of reticulocytes from the bone marrow in anemic patients [27]. We have previously shown that children with malarial anemia have reduced erythropoiesis (RPI<2.0) which increases with anemia severity [26–28, 32]. Comparison of the UM and SMA groups also revealed that white blood cell (WBC) counts were marginally elevated in children with SMA (*P*=0.08). Parasite density was significantly lower in children with SMA (*P*=0.03), while the proportions of children with high-density parasitemia (HDP: ≥10,000 parasites/μL) did not differ between the groups (*P*=0.11). The proportion of children with α-thalassemia (α^-3.7^/α^-3.7^), G6PD deficiency, and sickle-cell trait (HbAS) were comparable between the groups (*P*=0.42, *P*=0.45, and *P*=0.62, respectively).

**Table 1:** Demographic, clinical, and laboratory characteristics of the study participants

Characteristic	UM ( *n* = 718)	SMA (*n* = 165)	*P-*value
Sex			
Male, n (%)	356 (49.6)	79 (47.7)	0.72 ^a^
Female, n (%)	362 (50.4)	86 (52.3)	
Age (months)	12.2 (7.3–18.4)	10.0 (6.3–16.7)	**0.01** ^**b**^
Axillary temperature (°C)	37.6 (36.8–38.1)	38.0 (37.0–38.4)	**<0.02** ^**b**^
Hemoglobin (g/dL)	7.8 (6.2–9.1)	4.3 (3.7–4.7)	**<0.01** ^**b**^
RBC count (× 10^12^/μL)	3.7 (2.8–4.5)	2.9 (2.2–4.1)	**<0.01** ^**b**^
RDW	20.3 (18.1–23.0)	20.7 (18.0–23.3)	0.48^b^
MCHC	30.7 (29.6–32.3)	30.6 (29.5–32.3)	0.79 ^b^
RPI	0.5 (0.3–1.1)	0.5 (0.29–0.98)	0.42^b^
RPI <2.0, n (%)	605 (89.5)	136 (94.4)	0.07 ^a^
WBC count (× 10^3^/μL)	11.9 (9.0–15.9)	12.7 (9.5–17.0)	0.08 ^b^
Parasite density (per μL)	30,424.6 (7163.1-91,570.0)	24,180.0 (4605.7-71,849.7)	**0.03 ** ^**b**^
HDP (≥10,000 parasites/μL)	506 (70.7)	106 (64.2)	**0.11**
α-thalassemia, n (%)	130 (21.0)	24 (17.1)	0.42 ^a^
G6PD deficiency, n (%)	25 (4.0)	5 (3.5)	0.45^a^
Sickle cell trait, n (%)	102 (14.9)	21 (14.1)	0.62 ^a^

^a^Data are median (interquartile range, IQR) or n (%). UM [uncomplicated malaria, non-severe malarial anemia (Hb ≥5.0 g/dL)] and SMA [severe malarial anemia (Hb<5.0 g/dL)]; both with any density parasitemia. *RBC* red blood cell, *RDW* red cell distribution width, *MCHC* mean corpuscular hemoglobin concentration, *WBC* white blood cell and *HDP* high density parasitemia. The reticulocyte production index (RPI) was calculated as follows: RPI=reticulocyte index (RI)/maturation factor (MF), where RI=(reticulocyte count [%] x hematocrit [Hct]/0.36) and MF=b+(m)(x), where b=1, m=0.05, and x=(average normal population Hct - patient’s Hct) [27]; α-thalassemia data are for homozygous individuals (α^-3.7^); G6PD, glucose-6-phosphate dehydrogenase deficiency (G6PD)

^a^Statistical significance was determined by Chi-square analysis

^b^Statistical significance determined by Mann–Whitney U test. *P*-values ≤0.050 were considered significant, and are indicated in bold. n=Number

### IL-7 pilot GWAS and transcription factor binding site analysis

From the pilot GWAS, *IL7* emerged as a gene of interest based on statistical significance, and the fact that IL-7 is a hematopoietic growth factor which is largely unexplored in the pathogenesis of SMA [9]. Although the GWAS contained 32 SNPs encompassing the *IL7* haploblock (10.8 kb), we selected two SNPs (72194 T/C, rs2583759 and − 2440 A/G, rs7007634) for validation that contained high rankings on the *p*-value, MAFs (> 10%) in African populations, and contained variation that could impart functional changes based on *in-silico* analysis.

*In-silico* analysis of the − 2440 locus revealed that there is a potential TFBS that could potentially impact on the binding of TFs, and consequently transcriptional activity. In the simulated presence of the major allele A at the − 2440 locus, there is proposed binding for interferon regulatory transcription factor (IRF-3), c-E-twenty six transformation-specific-2 (c-Ets-2), activating enhancer binding protein 2 alpha (AP-2alphaA), and Nuclear Factor of Activated T Cells 1 (NF-AT1), while the simulated presence of the minor G allele, indicates binding for Early gene expression factor (Elk-1) and CCAAT/enhancer-binding protein beta (C/EBP beta) (Additional file 1: Table S1).

An A to G transition at the *IL7* -2440 locus results in loss of the NFAT1 binding site. NFAT1 is a transcription factor that induces expression of cytokines in T cells (e.g., IL-2, IL-3, IL-4, TNF-α and GM-CSF). Upon T-cell activation, NFAT1 is dephosphorylated by calcineurin and translocates to the nucleus [33].

### Distribution of *IL7* genotypes and haplotypes in the clinical groups

We assessed the distribution of the IL7 genotypes and the derived haplotypes between children presenting with UM and those with SMA. The overall genotypic frequencies at the 72194 T/C locus were; TT (n=391/883, 44.30%), TC (n=399/883, 45.20%) and CC (n=93/883, 10.50%) (Table 2). There was no significant departure from the Hardy Weinberg Equilibrium (HWE) observed for this variant in the overall population (χ^2^=0.04, *P*=0.83). For *IL7* (72194 T/C) there were 391 participants inheriting the homozygous wild type genotype (TT), of which 318 (81.30%) had UM (uncomplicated malaria), while 73 (18.70%) were in the SMA group. In addition, 399 study participants were heterozygous TC, of which 325 (81.50%) were in the UM and 74 (18.50%) in the SMA group. For this locus, the homozygous mutant CC, was inherited by 93 participants. These were distributed as follows; 75 (80.60%) were in UM while 18 (19.40%) were in the SMA groups, respectively. The genotypes in this locus were comparable between the two clinical categories (*P*=0.98). However, there was a significant departure from HWE at the *IL7* 72194 variant in both the UM (χ2=26.55, *P*<0.01) and SMA groups (χ2=6.78, *P*<0.01).

**Table 2:** Distribution of *IL7* genotypes and haplotypes in the clinical groups

Genetic Variants	Total (*n* = 883)	UM ( *n* = 718)	SMA (*n* = 165)	*P*-value
Genotypes				
*IL7* (72194 T/C)				
TT, n (%)	391 (44.30)	318 (44.30)	73 (44.20)	0.98
TC, n (%)	399 (45.20)	325 (45.30)	74 (44.80)	
CC, n (%)	93 (10.50)	75 (10.40)	18 (10.90)	
*IL7* (−2440 A/G)				
AA, n (%)	733 (83.00)	601 (83.70)	132 (80.00)	0.51
AG, n (%)	142 (16.10)	111 (15.50)	31 (18.80)	
GG, n (%)	8 (0.90)	6 (0.80)	2 (1.20)	
Haplotypes				
TA				
0	99 (11.20)	79 (11.00)	20 (12.10)	0.68
1	784 (88.70)	639 (89.00)	145 (87.90)	
TG				
0	821 (93.00)	674 (93.90)	147 (89.10)	**0.03**
1	62 (7.00)	44 (6.10)	18 (10.90)	
THAT				
0	468 (53.10)	382 (53.20)	86 (52.10)	0.80
1	415 (46.90)	336 (46.80)	79 (47.90)	
CG				
0	792 (89.70)	642 (89.40)	150 (90.90)	0.57
1	91 (10.30)	76 (10.60)	15 (9.10)	

Data are presented as proportions [n, (%)] of *IL7* genotypes (72194 T/C and − 2440A/G) and haplotypes (TA, TG, CA, and CG). UM [uncomplicated malaria, non-severe malarial anemia (Hb ≥5.0 g/dL)] and SMA [severe malarial anemia (Hb < 5.0 g/dL)]; both with any density parasitemia. Non-carriers (0) and carriers (1). Statistical significance was determined by Chi-square analysis comparing UM versus SMA

The overall genotypic frequencies at the − 2440 A/G locus were: AA (*n* = 733/883, 83.00%), AG (*n* = 142/883, 16.10%), and GG (*n* = 8/883, 0.90%). No significant departure from Hardy Weinberg Equilibrium (HWE) was observed for this variant in the overall population (χ2 = 0.01, *P* = 0.81). The distribution in the *IL7* (− 2440 A/G) was as follows: homozygous wild type (AA) was inherited by 733 study participants distributed as 601 (82.00%) in UM and 132 (18.00%) in the SMA group. The heterozygous AG was inherited by 142 participants, of which 111 (78.20%) and 31 (21.80%) were in UM and SMA groups, respectively. In addition, the homozygous mutant was inherited by 8 study participants, of which 6 (75.00%) were in UM and 2 (25.00%) were in SMA clinical category. The distribution of the genotypes at this locus was comparable between UM and SMA (*P* = 0.51). There was significant departure from HWE at the *IL7*–2440 A/G locus in both the UM (χ2 = 26.60, *P* < 0.01) and SMA (χ2 = 6.60, *P* = < 0.01) groups.

Following analyses of the individual variants, haplotypes of 72194 T/C and − 2440 A/G locus were constructed using HPlus software (version 2.5). The following haplotypes were derived: TA, TG, CA and CG. The TA haplotype was inherited by 784 study participants, of which, 639 (81.50%) were in UM and 145 (18.50%) were in the SMA group. The distribution of this haplotype was comparable between UM and SMA groups (*P* = 0.68). The TG haplotype was inherited by 62 participants, 44 (71.00%) were in UM and 18 (29.00%) were in SMA group. The proportion of TG haplotype was higher in the UM relative to the SMA clinical category (*P* = 0.03). The CA haplotype was inherited by 415 participants, of these, 336 (81.00%) was in the UM while 79 (19.00%) was in the SMA group. The proportion of CA haplotype was comparable between the clinical categories (*P* = 0.80). The CG haplotype was inherited by 91 study participants, 76 (83.5%) was in UM and 15 (16.5%) was in SMA group. The proportion of CG haplotype was comparable between the two clinical categories (*P* = 0.57). There was a weak magnitude of linkage disequilibrium (LD) for the *IL7* SNPs 72194/− 2440 (D’ = 0.11; r^2^ < 0.01).

### Susceptibility to SMA and inefficient erythropoiesis (RPI < 2.0)

To determine the impact of the *IL7* variants (genotypes and haplotypes) on susceptibility to SMA and inefficient erythropoiesis, we performed binary logistic regression analyses in a model controlling for the potential confounding effects of age, gender, HIV-1 and bacteremia status, G6PD deficiency, Hb phenotypes, and α-thalassemia status. These results are summarized in Table 3. Relative to 72194 TT carriers, neither TC (OR = 0.93; 95% CI = 0.61–1.42; *P* = 0.73) nor CC (OR = 1.34; 95% CI = 0.69–2.58; *P* = 0.39) influenced susceptibility to SMA. Similarly, relative to − 2440 AA inheritance, AG (OR = 1.24; 95% CI = 0.74–2.09; *P* = 0.41) and GG carriers (OR = 0.80; 95% CI = 0.09–7.13; *P* = 0.84) did not have altered susceptibility to SMA, suggesting that potential ablation of the NFAT1 binding site through carriage of the G allele did not functionally influence disease outcomes. In addition, we determined the association between the derived haplotypes and susceptibility to SMA. Our results show that relative to non-haplotype carriers, TA (OR = 0.70; 95% CI = 0.38–1.29; *P* = 0.26), TG (OR = 1.29; 95% CI = 0.61–2.71; *P* = 0.51), CA (OR = 1.04; 95% CI = 0.69–1.56; *P* = 0.86), and CG (OR = 0.94; 95% CI = 0.48–1.85; *P* = 0.86) haplotypic carriage had no impact on susceptibility to SMA.

**Table 3:** Relationship between genotypes/haplotypes and susceptibility to SMA and Inefficient Erythropoiesis (RPI<2)

SMA				RPI < 2.0		
Genetic Variants	OR	95% CI	*P*-value	OR	95% CI	*P*-value
Genotypes						
*IL7* (72194 T/C)						
TT ^a^	0.80	0.47–1.34	0.39	0.84	0.37–1.90	0.67
TC	0.93	0.61–1.42	0.73	1.90	1.09–3.30	**0.02**
CC	1.34	0.69–2.58	0.39	5.14	1.20–21.99	**0.03**
*IL7* (−2440 A/G)						
AA	Ref			Ref		
AG	1.24	0.74–2.09	0.41	1.38	0.63–3.03	0.42
GG	0.80	0.09–7.13	0.84	–	–	–
Haplotypes						
TA	0.70	0.38–1.29	0.25	0.24	0.06–1.21	**0.05**
TG	1.29	0.61–2.71	0.50	1.17	0.40–3.44	0.78
THAT	1.04	0.69–1.56	0.85	1.90	1.10–3.30	**0.02**
CG	0.94	0.48–1.85	0.86	1.66	0.57–4.80	0.35

Data are presented as odd ratios (OR) and 95% confidence interval (CI) determined by bivariate logistic regression analyses controlling for age, sex, HIV-1 and bacteremia status, α-thalassemia, G6PD deficiency, and sickle-cell status. *P*-values ≤0.050 were considered significant (indicated in bold font). Parasitemic children were categorized into UM (uncomplicated malaria, Hb ≥ 5.0 g/dL, *n* = 718) and SMA (severe malarial anemia, Hb < 5.0 g/dL, *n* = 165), and RPI ≥ 2 (*n* = 64) and RPI < 2.0 (*n* = 611)

^a^To establish the association between TT genotype and SMA and reduced erythropoiesis, the combined TC/CC were used as reference

Since altered erythropoietic responses are central to the pathogenesis of SMA [34], we examined the association between genotypes/haplotypes and inefficient erythropoiesis (RPI < 2.0) in the overall population. Inheritance of the 72194 TC (OR = 1.90; 95% CI = 1.09–3.30; *P* = 0.02) and CC (OR = 5.14; 95%CI = 1.20–21.99; *P* = 0.03) genotypes enhanced susceptibility to inefficient erythropoiesis. However, relative to − 2440 AA carriage, inheritance of the AG (OR = 1.39; 95% CI = 0.63–3.03; *P* = 0.42) genotype did not significantly influence susceptibility to inefficient erythropoiesis. The sample size in the clinical groups for determining susceptibility to inefficient erythropoiesis in the GG group was too small for logistic regression analysis. The relationship between haplotypes and inefficient erythropoiesis was also determined. Inheritance of the TA haplotype was associated with moderate protection against inefficient erythropoiesis (OR = 0.24; 95% CI = 0.06–1.21; *P* = 0.05), whereas individuals with the CA haplotype were at an increased risk of inefficient erythropoiesis (OR = 1.90; 95% CI = 1.10–3.30; *P* = 0.02). Carriage of the TG (OR = 1.17; 95% CI = 0.40–3.44; *P* = 0.78) and CG (OR = 1.66; 95% CI = 0.57–4.80; *P* = 0.35) haplotypes were not associated with altered susceptibility to inefficient erythropoiesis. These observations were supported by Cochran-Armitage trend test for inefficient erythropoiesis (CA > TA > CG; *P* < 0.01).

In addition, results from the hierarchical linear regression analyses revealed that only the 72194 T/C genotype significantly predicted RPI. Block 1 was R^2^ = 0.01, *P* = 0.15 and block 2 was R^2^ = 0.02, *P* = 0.04. The 72194 T/C genotype had a β-weight of 0.11, semipartial r^2^ of 0.09, and *P* = 0.02.

## Discussion

Severe malarial anemia (SMA) is a major cause of morbidity and mortality in western Kenya [2]. The pathogenesis of SMA in holoendemic regions is multifactorial and includes lysis of infected and uninfected RBCs**,** splenic sequestration of RBCs**,** dyserythropoiesis and bone marrow suppression**,** co-infections with bacteremia, HIV-1, and hookworm**,** and chronic transmission of malaria [28, 35–37].

Our previous investigations demonstrated that variations in immune mediator genes, such as IL-1β, IL-12, IL-10, IL-13, IL-18, MIF, and SCGF, influence susceptibility to SMA [3–8, 38]. Although the candidate gene approach can be a viable means of uncovering the molecular pathways that influence malaria disease outcomes, unbiased approaches, such as global genomic characterization can identify both novel and known genetic variants associated with complex human phenotypes. As such, we performed a GWAS and whole-transcriptome profiling in a case-control study of children infected with falciparum malaria that were stratified into polarized phenotypes: SMA (cases, avg. Hb = 4.10 g/dL) and UM (controls, avg. Hb = 10.80). Among a prioritized list of genes for further investigation, *IL7* emerged as a target for further evaluation (unpublished). On the basis of these findings, we selected variants in *IL7* (72194 T/C, located in the intronic region and − 2440 A/G located in promoter region) and explored their association with SMA and inefficient erythropoiesis.

The importance of *IL7* in conditioning inflammatory disease outcomes has been previously described [19–21]. To the best of our knowledge, this is the first pediatric study to examine the association between the *IL7* polymorphisms and susceptibility to these severe disease outcomes in malaria. First, we compared the variant frequencies of the two loci separately and found that the prevalence of individual genotypes at both loci were comparable between the UM and SMA groups. In addition, the single-locus minor allele frequencies (0.33 for 72194C and 0.09 for -2440G) in this population were the same as those for the Yoruban Nigerian population in the HapMap (i.e., 0.33 and 0.09, respectively). These results suggest that the investigated alleles remain largely unchanged over time in different African ethnic groups. However, since the overall genotypic frequencies for the 72194 T/C and -2440A/G SNPs in the UM and SMA groups departed from HWE, it is feasible that population selection pressures from malaria may be responsible for this observation.

To date, only one study has reported an association between *IL7* genetic variation and malaria. This study, in pregnant women from Mozambique, revealed an association between *IL7* SNPs (rs2583764 and rs2583762, 33 kb apart) and increased susceptibility to placental malaria [22]. These two SNPs are in close proximity to SNP 72194 T/C (rs2583759) genotyped in the present study. This particular SNP (72194 T/C), along with additional SNPs in the same haploblock (rs2583760, rs2583764 and rs6993386), were associated with susceptibility to osteoarthritis in the Chinese Han population [20]. Although we observed significant associations between genotypes and SMA in our GWAS pilot data, this was not replicated in the validation sample. Thus, the signal for SMA in the pilot study was a false-positive and may be attributed to the relatively small sample size in the pilot GWAS study. Alternatively, the pilot study examined children with extreme phenotypes. By including children in the validation study that were parsed into two groups above and below Hb of 5.0 g/dL, there are many children with low Hbs, yet slightly above 5.0 g/dL in the UM group. This may have weakened the ability to identify a significant signal, and is consistent with the reality that there is not a clear-cut clinical difference between Hb levels of 6.0 vs. 5.0 g/dL. Results presented here indicate that carriage of the C allele at *IL7* 72194 loci significantly increases susceptibility to inefficient erythropoiesis (i.e., RPI < 2.0) in both heterozygous and homozygous carriers. In addition, the trend of reduced susceptibility (TT < TC < CC) in cases with inefficient erythropoiesis was maintained. However, variation at *IL7* 72194 was not significantly associated with susceptibility to SMA, although carriage of the CC allele trended towards increased risk. This finding suggests that the association between *IL7* 72194 C allele carriage and erythrocyte production are more closely linked than the phenotypic expression of SMA in which reduced Hb concentrations are a central feature. This result may not be unexpected since inefficient erythropoiesis is an overlapping feature of both UM (89.50%) and SMA (94.40%), albeit slightly higher in SMA. Although a primary cause of SMA in the region is suppression of erythropoiesis, hemolysis of parasitized and non-parasitized RBCs also contributes to the reduced Hb levels [2]. Consistent with the importance of inefficient erythropoiesis in SMA, there was a significant relationship between RPI<2.0 and SMA (Additional file 2: Table S2). Further, we did not find any significant association between *IL7–*2440A/G and either SMA or inefficient erythropoiesis.

Since haplotypic carriage can provide additional insight, beyond analysis of the individual loci, we constructed *IL7* haplotypes from the two SNPs (72194 T/C and -2440A/G). Our previous results demonstrated that haplotypes are highly informative allelic markers for identifying associations with disease outcomes, not identifiable with single polymorphisms [3–7, 39]. Findings from this study show that carriage of the TA haplotype (72194 T/−2440A) was significantly associated with a reduced risk of inefficient erythropoiesis. This finding reflects the fact that carriage of the wild-type alleles at the two loci each trended towards better erythropoietic responses. Conversely, the relationship between carriage of the C allele at *IL7* 72194 and inefficient erythropoiesis was reflected in the haplotypes in which the CA haplotype was significantly associated with an RPI < 2.0, and the CG haplotype was associated with a non-significant increase in the risk of inefficient erythropoiesis. Similar to the findings for the individual genotypes, none of the haplotypes were associated with susceptibility to SMA. However, the directionality of the risk profiles for SMA and inefficient erythropoiesis were consistent. Since there were not enough children in the current study for which circulating IL-7 levels were available, we could not explore the functional association between the *IL7* variants and cytokine production. Previous investigations from our laboratory demonstrated that circulating levels of IL-7 were reduced in children with SMA, but were not significant predictors of Hb concentrations [24]. Moreover, IL-7 promotes T cell-dependent activation of monocyte/macrophages which, in turn, release a number of cytokines (i.e., IL-1, IL-3, IL-6, IL-8, GM-CSF, MIP-1β, TNF-α, and IFN-γ) whose dysregulation is associated with SMA [2, 24, 40–43]. Additionally, since IL-7 induces erythropoiesis, reduced levels in children with SMA may contribute to delayed and/or inappropriate erythroid development [42].

This study presents the first report on the association between genotypes and haplotypes of *IL7* (72194 T/C and -2440A/G) and disease outcomes in children with severe malaria: profoundly low Hb levels and inefficient erythropoiesis. Results presented here suggest that the selected variants are associated with erythropoietic responses. Additional studies are required to determine if the explored *IL7* variants functionally alter IL-7 production. It will also be important to determine if products derived from malarial parasite (e.g., antigens, soluble parasitic molecules, and hemozoin) can alter IL-7 production. Investigations in our laboratory are currently exploring these additional experiments in the context of IL-7 and other inflammatory mediators.

We, however, want to note the following limitations of this study. There was a small sample size in the pilot GWAS (*n* = 144) and moderate sample size in the validation set (*n* = 883). Given the relatively low frequency of the G allele at the − 2440 locus, the haplotype analyses for this locus may be underpowered.

## Conclusion

Results presented here suggest that variation in *IL7* is associated with altered erythropoietic responses in children with uncomplicated vs. severe falciparum malaria. These findings have relevance to the development of SMA in children with malaria since inappropriate erythropoiesis is a central physiological feature contributing to development of SMA.

## Additional files

Additional file 1: **Table S1.** Transcription factor binding analysis. Transcription factor binding analysis of the *IL-7*2440 A/G (rs7007634)] and amino acid change for the [72194 T/C (rs2583759). (DOCX 16 kb)

Additional file 2: **Table S2.** Regression analysis. Relationship between reduced erythropoiesis and SMA. (DOCX 14 kb)

Additional file 3: Questionnaire. Demographic and clinical data collected at enrollment. (XLS 494 kb)

## Data Availability

The datasets used and/or analyzed in the current manuscript are available from the corresponding author on reasonable request.

## Abbreviations

CI: Confidence interval
G-CSF: Granulocyte colony stimulating factor
GM-CSF: Granulocyte Monocyte colony stimulating factor
GWAS: Genome-wide Association Study
Hb: Hemoglobin
HWE: Hardy Weinberg Equilibrium
MIP-1β: Macrophage inflammatory protein-1β
OR: Odds ratio
RBCs: Red blood cells
RPI: Reticulocyte production index
SCRH: Siaya County Referral Hospital
High school: Severe malarial anemia
SNPs: Single nucleotide polymorphisms
SSD: Sickle-cell disease
TFBS: Transcription factor binding site
TNF-α: Tumor necrosis factor alpha
