# Supplementary material for: Genetic variation in interleukin-7 is associated with a reduced erythropoietic response in Kenyan children infected with Plasmodium falciparum
Source: BMC Med Genet. 2019 Aug 16;20:140. doi: 10.1186/s12881-019-0866-z (PMC6698010; doi:10.1186/s12881-019-0866-z)
Supplement: Supplementary file 1 — Table S1. Transcription factor binding analysis. Transcription factor binding analysis of the IL-72440 A/G (rs7007634)] and amino acid change for the [72194 T/C (rs2583759). (DOCX 16 kb) [file 12881_2019_866_MOESM1_ESM.docx]

**Additional file 1: Table S1: Transcription factor binding analysis of the *IL-7* 2440 A/G (rs7007634)] and amino acid change for the [72194T/C (rs2583759).**

| **Gene** | **SNP** | **Major allele binding factors** | **Minor allele binding factors** |
| --- | --- | --- | --- |
| *IL-7* | -2440 A/G (rs7007634) | **A**  IRF-3, c-Ets-2, AP-2alphaA, and NF-AT1 | **G**  Elk-1 and C/EBPbeta |
|  |  | **Major allele protein** | **Minor allele protein** |
| *IL-7* | 72194T/C (rs2583759) | **T**  Serine | **C**  Leucine |

*In-silico* transcription factor binding site (TFBSs) analyses were performed on the region encompassing the promoter variant using TRANSFAC to determine potential gain or loss of binding for transcription factors (TFs) due to SNP variation. IRF-3, interferon regulatory transcription factor; c-Ets-2, c-E-twenty six transformation-specific-2; AP-2alphaA, activating enhancer binding protein 2 alpha; NF-AT1, Nuclear Factor of Activated T Cells 1; Elk-1, Early gene expression factor; and C/EBP beta, CCAAT/enhancer-binding protein beta.
