# Supplementary material for: Genetic variation in interleukin-7 is associated with a reduced erythropoietic response in Kenyan children infected with Plasmodium falciparum
Source: BMC Med Genet. 2019 Aug 16;20:140. doi: 10.1186/s12881-019-0866-z (PMC6698010; doi:10.1186/s12881-019-0866-z)
Supplement: Supplementary file 2 — Table S2. Regression analysis. Relationship between reduced erythropoiesis and SMA. (DOCX 14 kb) [file 12881_2019_866_MOESM2_ESM.docx]

**Additional file 2: Table S2: Relationship between reduced erythropoiesis and SMA.**

| **SMA** | | | |
| --- | --- | --- | --- |
| **Clinical category** | **OR** | **95%CI** | ***P*-value** |
| RPI>2 | Ref |  |  |
| RPI<2 | 2.65 | 1.03-6.84 | **0.04** |

Data are presented as odd ratios (OR) and 95% confidence interval (CI) determined by bivariate logistic regression analyses controlling for age, sex, HIV-1 and bacteremia status, α-thalassemia, G6PD deficiency, and sickle-cell status. *P*-values ≤0.050 were considered significant (indicated in bold font). Parasitemic children were categorized into UM (Hb≥5.0 g/dL, n=718) and SMA (Hb<5.0 g/dL, n=165), and RPI≥2 (n=64) and RPI<2.0 (n=611).
